# Analysis of Oral versus Long-acting Injectable Antipsychotics in the Maintenance of Schizophrenia

**DOI:** 10.1192/j.eurpsy.2022.820

**Published:** 2022-09-01

**Authors:** O. Vasiliu

**Affiliations:** Dr. Carol Davila University Emergency Central Military Hospital, Psychiatry, Bucharest, Romania

**Keywords:** schizophrénia, therapeutic adherence, Antipsychotics

## Abstract

**Introduction:**

A debate regarding the comparative efficacy and tolerability of oral and long-acting injectable antipsychotics (LAIs) in patients diagnosed with schizophrenia is still open in the mind of clinicians. While the adherence is intuitively improved by the LAIs, the acceptance of this treatment is not always good.

**Objectives:**

To conduct a literature review in order to find the data about the comparative efficacy of oral and LAI antipsychotics in schizophrenia, during the maintenance phase.

**Methods:**

A literature review was performed through the main electronic databases (PubMed, CINAHL, SCOPUS, EMBASE) using the search paradigm “schizophrenia” AND “maintenance treatment” AND “oral antipsychotics” OR “long-acting injectable antipsychotics”. All papers published between January 2000 and August 2021 were included.

**Results:**

Based on the reviewed clinical trials (n=37), LAI antipsychotics are associated with an efficacy and tolerability profile similar to or slightly superior to the oral formulation. This is confirmed for both typical and atypical antipsychotics that have been detected by this review. The main advantage of the LAIs is their superior therapeutic compliance and the possibility of early detection for non-adherent patients. However, not all data are unanimously supporting this perspective: while observational trials favor LAI antipsychotics, randomized trials included in the meta-analyses do not detect significant differences between the two types of formulations.

**Conclusions:**

LAIs are associated with superior adherence, but their overall efficacy and tolerability are only slightly superior to those of the oral equivalents. Moreover, there are differences in the results related to the methodology of the trials, therefore data should be interpreted with care.

**Disclosure:**

No significant relationships.

